# Distinct molecular subtypes of uterine leiomyosarcoma respond differently to chemotherapy treatment

**DOI:** 10.1186/s12885-017-3568-y

**Published:** 2017-09-11

**Authors:** Yang An, Shuzhen Wang, Songlin Li, Lulu Zhang, Dayong Wang, Haojie Wang, Shibai Zhu, Wan Zhu, Yongqiang Li, Wenwu Chen, Shaoping Ji, Xiangqian Guo

**Affiliations:** 1 Department of Biochemistry and Molecular Biology, Joint National Laboratory for Antibody Drug Engineering, Institute of Biomedical Informatics, Medical School, Henan University, Kaifeng, 475004 China; 2 Cell signal transduction Laboratory, Henan University, Kaifeng, 475004 China; 3 Department of Neurology, The First Affiliated Hospital of Henan University, Kaifeng, 475001 China; 4 Department of Nuclear Medicine, The First Affiliated Hospital of Henan University, Kaifeng, 475001 China; 5 Department of Orthopedic Surgery, Peking Union Medical College, Chinese Academy of Medical Science, Beijing, 100730 China; 6 Department of Anesthesia and Perioperative Care, University of California, San Francisco, San Francisco, CA 94110 USA; 7 Department of Preventive Medicine, Medical School, Henan University, Kaifeng, 475004 China; 8 Institute of Environmental Medicine, Henan University, Kaifeng, 475004 China

**Keywords:** Uterine leiomyosarcoma, Molecular subtype, Molecular signature, Gene expression pattern, Subtype-specific treatment

## Abstract

**Background:**

Uterine leiomyosarcoma (ULMS) is an aggressive form of soft tissue tumors. The molecular heterogeneity and pathogenesis of ULMS are not well understood.

**Methods:**

Expression profiling data were used to determine the possibility and optimal number of ULMS molecular subtypes. Next, clinicopathological characters and molecular pathways were analyzed in each subtype to prospect the clinical applications and progression mechanisms of ULMS.

**Results:**

Two distinct molecular subtypes of ULMS were defined based on different gene expression signatures. Subtype I ULMS recapitulated low-grade ULMS, the gene expression pattern of which resembled normal smooth muscle cells, characterized by overexpression of smooth muscle function genes such as *LMOD1*, *SLMAP*, *MYLK*, *MYH11.* In contrast, subtype II ULMS recapitulated high-grade ULMS with higher tumor weight and invasion rate, and was characterized by overexpression of genes involved in the pathway of epithelial to mesenchymal transition and tumorigenesis, such as *CDK6*, *MAPK13* and *HOXA1*.

**Conclusions:**

We identified two distinct molecular subtypes of ULMS responding differently to chemotherapy treatment. Our findings provide a better understanding of ULMS intrinsic molecular subtypes, and will potentially facilitate the development of subtype-specific diagnosis biomarkers and therapy strategies for these tumors.

**Electronic supplementary material:**

The online version of this article (10.1186/s12885-017-3568-y) contains supplementary material, which is available to authorized users.

## Background

Uterine leiomyosarcoma (ULMS) is a type of malignant soft tissue tumors showing distinctive morphologic features and molecular signatures [1]. ULMS has poor prognosis and high recurrence rate [2–4]. Currently, the treatment of ULMS is mostly by surgery with some adjuvant therapies, such as cytotoxic chemotherapy and radiotherapy [5–7]. Due to the complex molecular heterogeneity of ULMS and unavailability of targeted therapeutic methods, the five-year survival rate of ULMS is still low [8]. By gene expression profiling methods, a number of malignant tumors, including breast cancer, gastric cancer, and uterine carcinosarcoma [9–13], have been categorized into different molecular subtypes. Based on the subtype information, patients may be able to receive better diagnosis and more effective therapeutic options [14]. Thus, it is important to classify molecular subgroups of ULMS, which may provide a better understanding of disease mechanism and guide future precision treatment. Previously, we analyzed leiomyosarcoma (LMS) cases from uterine and extra-uterine sites to classify molecular subtypes [15]. Nevertheless, the treatments for LMS were different regarding LMS locations, which were between uterine and extra-uterine LMS patients in clinical practice. Italiano et al. demonstrated the molecular heterogeneity of LMS from extra-uterus by genetic profiling [16]. But the molecular heterogeneity of ULMS was less investigated until now.

In this study, by analyzing gene expression data sets, we identified and defined two molecular subtypes of ULMS, each of which presents subtype-specific gene expression patterns. Genes and pathways enriched in subtype I ULMS were associated with smooth muscle function, while genes and pathways involved in epithelial to mesenchymal transition (EMT) and tumorigenesis were enriched in subtype II ULMS. Our findings will provide a better understanding of ULMS pathogenesis and facilitate the development of more effective and individualized therapies.

## Methods

### Bioinformatic analyses and immunohistochemistry staining

To identify the molecular subtypes of ULMS, we collected and analyzed expression profile data sets from TCGA database and determined the optimal number of molecular subtypes of ULMS by Consensus Clustering (R package, ConsensusClusteringPlus [17]). The accuracy of subtype assignments was evaluated by Silhouette analysis (R package cluster [18]). The subtype-specific gene expression patterns were investigated by Gene Set Enrichment Analysis (GSEA) and Significance Analysis of Microarrays (SAM-seq). To identify the pathways that enriched in each subtype, the KEGG pathway analysis was performed online (https://david.ncifcrf.gov/). Cluster 3.0 and TreeView were used to perform hierarchical clustering to view the top 500 significantly over-expressed genes from each subtype. To develop subtype specific diagnostic biomarkers, genes overexpressed in subtype I (*LMOD1*, 1:20, Sigma, CAT#HPA028325) and subtype II (*ARL4C*, 1:120, Sigma, CAT#HPA028927) ULMS were selected for immunohistochemistry staining (IHC) based on SAM-seq result and the antibody availability. The procedure and scoring of IHC were performed as described previously [15].

### Statistical analyses

Statistical significance was assessed by the chi-square and Fisher exact tests. For all statistical analyses, *p* value less than 0.05 was considered statistically significant.

## Results

### Consensus clustering of gene expression profiles revealed two molecular subtypes of uterine leiomyosarcoma

**Fig. 1:**
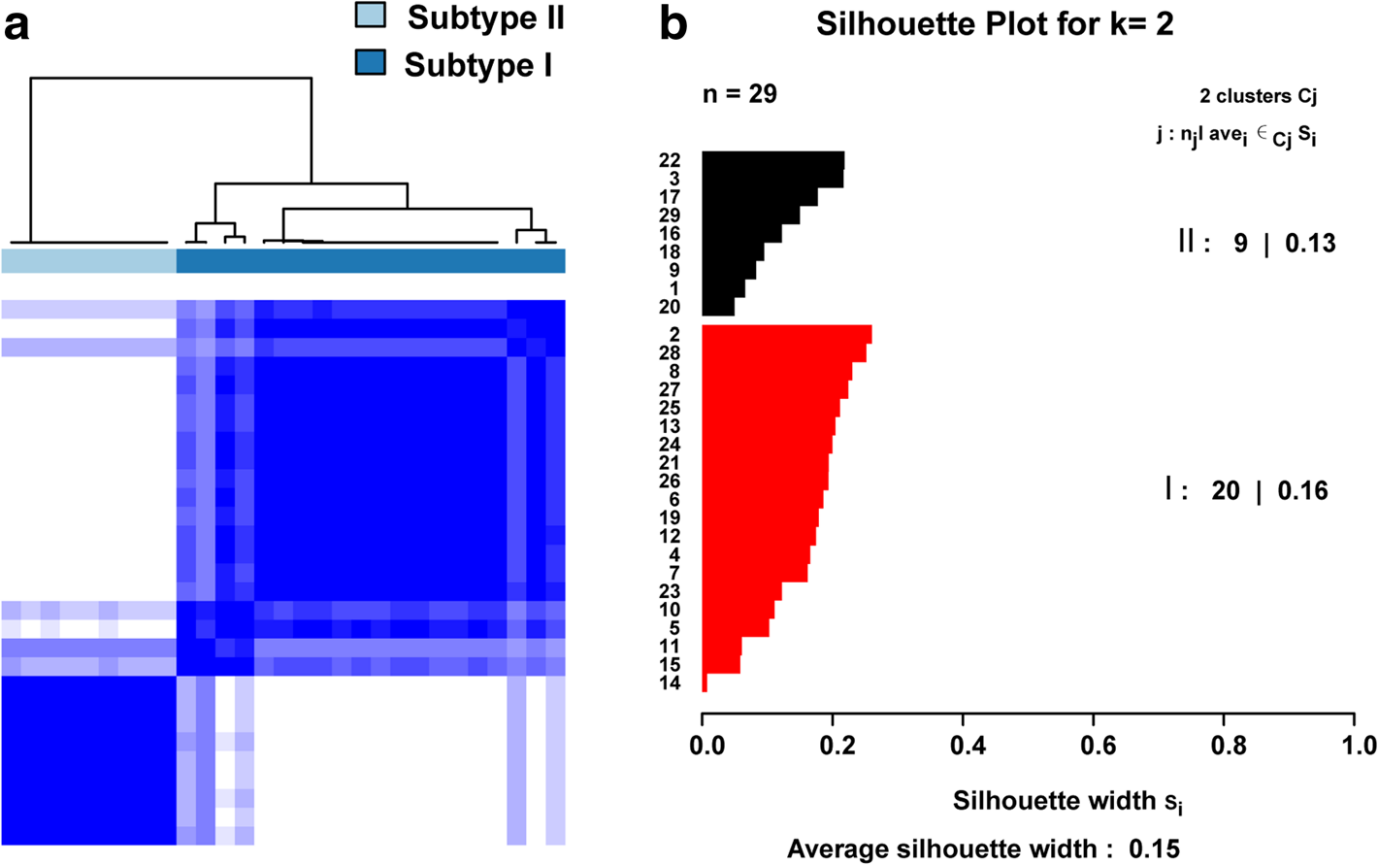
Identification of two distinct molecular subtypes of ULMS. **a** Consensus clustering reveals two distinct molecular subtypes of ULMS. Each column corresponds to a case of ULMS. **b** Silhouette analysis validates the subtype assignments from consensus clustering

Level 3 RNAseq expression data of 29 ULMS cases were collected from The Cancer Genome Atlas (TCGA) and used to determine the molecular heterogeneity of ULMS by consensus clustering (Fig. 1a), a method that estimates cluster stability by iterative resampling of genes and samples [17]. The consensus clustering demonstrated that two subtypes were the optimal number for ULMS, as indicated by the empirical cumulative distribution plots, showing the greatest increase in the area under CDF curve (Additional file 1: Figure S1A and B). Next, the confidence of subtype assignment from Consensus Clustering was evaluated by silhouette analysis (Fig. 1b), which showed that all cases from both subtypes have a positive silhouette value, confirming the two molecular ULMS subtypes.

### Clinicopathologic features of ULMS molecular subtypes

Next, we compared the clinicopathologic features between subtype I and subtype II ULMS patients. As shown in Table 1, the ULMS subtype is significantly associated with clinical treatment response. Specifically, subtype I patients were significantly more responded to chemotherapy treatment than subtype II. However, there is no significant association between molecular subtypes with other clinicopathologic characteristics, including tumor weight, metastasis status, invasion and necrosis (Table 1).

**Table 1:** Clinicopathologic characteristics (*N* = 29)

	patients, *n* (%)	Subtype I	Subtype II	*p* value
Age (year)				
Mean	56	54	60	
Range	35–73	35–73	41–71	
Race				
White	20	14	6	
Black or African American	4	2	2	
Asian	1	1	0	
Unknown	4	3	1	
Tumor weight				0.4
Mean	341.83	303.8	426.33	
Range	55–1100	58–1100	55–1077	
Treatment best response				0.04
Complete Response	8(27%)	6	2	
Partial Response	2(7%)	0	2	
Clinical Progressive Disease	4(14%)	4	0	
Unknown	15(52%)	10	5	
Metastatic disease confirmed				0.18
YES	10 (34%)	5	5	
NO	11 (38%)	9	2	
Unknown	8 (28%)	6	2	
Contiguous organ invaded				0.24
YES	4(14%)	2	2	
NO	8 (28%)	7	1	
Unknown	17 (58%)	11	6	
Necrosis percent				0.52
Mean	20.33	23.5	14	
Range	0–50	1–50	0–33	

* *p* < 0.05

### Distinct molecular subtypes of ULMS have different gene expression patterns

**Fig. 2:**
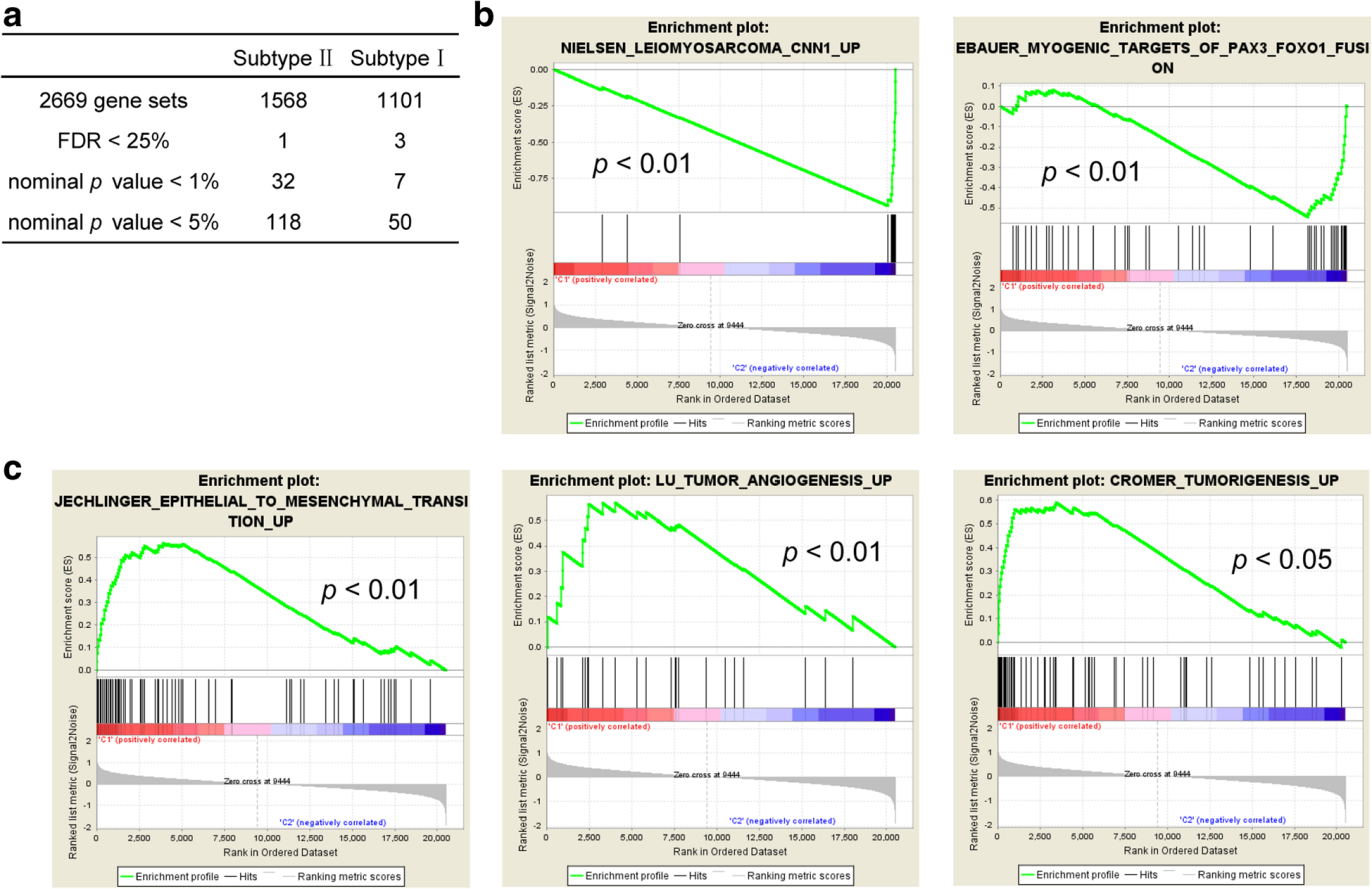
Different gene sets enriched in distinct molecular subtypes. **a** The summary of GSEA results. **b** and **c** The gene sets enriched in subtype I and subtype II, respectively. Permutation = 1000, *p* < 0.05

**Fig. 3:**
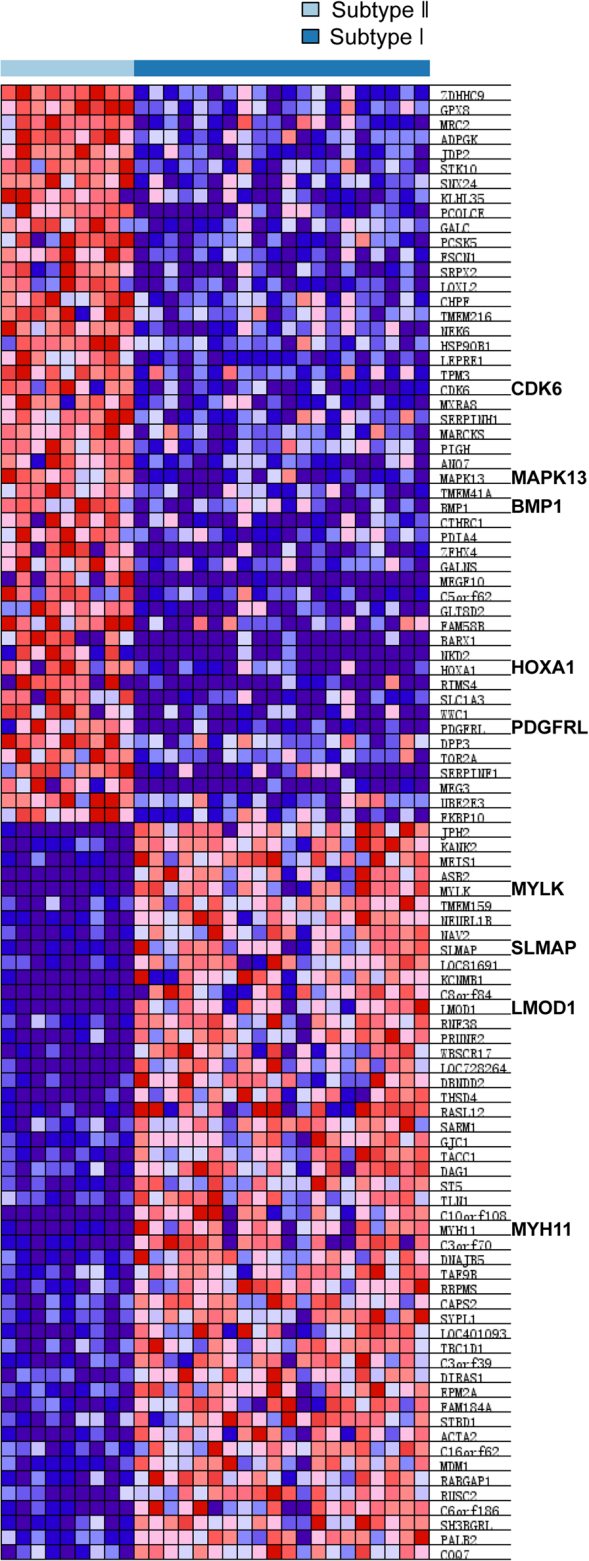
Different gene expression signatures enriched in distinct molecular subtypes. Subtype I and subtype II ULMSs have different gene expression signatures revealed by GSEA. Each row denotes a gene and each column corresponds to a case of ULMS. Red, over-expressed genes; Blue, down-expressed genes

We next investigated the subtype-specific gene expression patterns of ULMS by Gene Set Enrichment Analysis (GSEA) [19]. GSEA analysis showed that 2669 gene sets were enriched. Among the 2669 gene sets, 1568 gene sets were over-expressed in subtype II while the other 1101 gene sets were over-expressed in subtype I (Fig. 2a). Gene sets associated with leiomyosarcoma and myogenic targets were enriched in subtype I (Fig. 2b), whereas gene sets involved in EMT and tumorigenesis were enriched in subtype II (Fig. 2c). Interestingly, genes over-expressed in subtype II were also associated with cell cycle, proliferation, organ development and tumorigenesis. These genes include *CDK6*, *BMP1*, *MAPK13*, *PDGFRL* and *HOXA1* (Fig. 3). Subtype I ULMS was enriched with genes involved in smooth muscle function (Fig. 3), including *LMOD1*, *SLMAP*, *MYLK*, and *MYH11,* all of which are the smooth muscle-specific markers [20–22].

To further explore the different gene expression patterns of the two subtypes of ULMS, we performed Significance Analysis by SAM-seq. Our results showed that 1947 genes were significantly differently expressed between two subtypes. Among these genes, 1050 were over-expressed in subtype II ULMS while 897 genes were over-expressed in subtype I. Next, we analyzed the top 500 over-expressed genes in each subtype by hierarchical clustering. As the heatmap shows, those genes were significantly over-expressed in subtype I and subtype II, respectively (Additional file 2: Figure S2). Consistent with the GSEA results, the pathways enriched in subtype I were associated with smooth muscle function, such as vascular smooth muscle contraction, calcium signaling pathway, and regulation of actin cytoskeleton (Table 2). Pathways involved in tumorigenesis associated with subtype II, such as pathways in cancer, TGF-β and Hedgehog signaling pathway (Table 2).

**Table 2:** Pathways enriched in each molecular subtype

	Pathways	*p* Value
Subtype I	Vascular smooth muscle contraction	7.2E-08
	Focal adhesion	0.00021
	Calcium signaling pathway	0.00497
	Regulation of actin cytoskeleton	0.00702
Subtype II	Ribosome	1.7E-05
	Hedgehog signaling pathway	1.28E-02
	Pathways in cancer	0.02433
	TGF-beta signaling pathway	0.03385

### Immunohistochemistry staining of ULMS subtype specific biomarkers

**Fig. 4:**
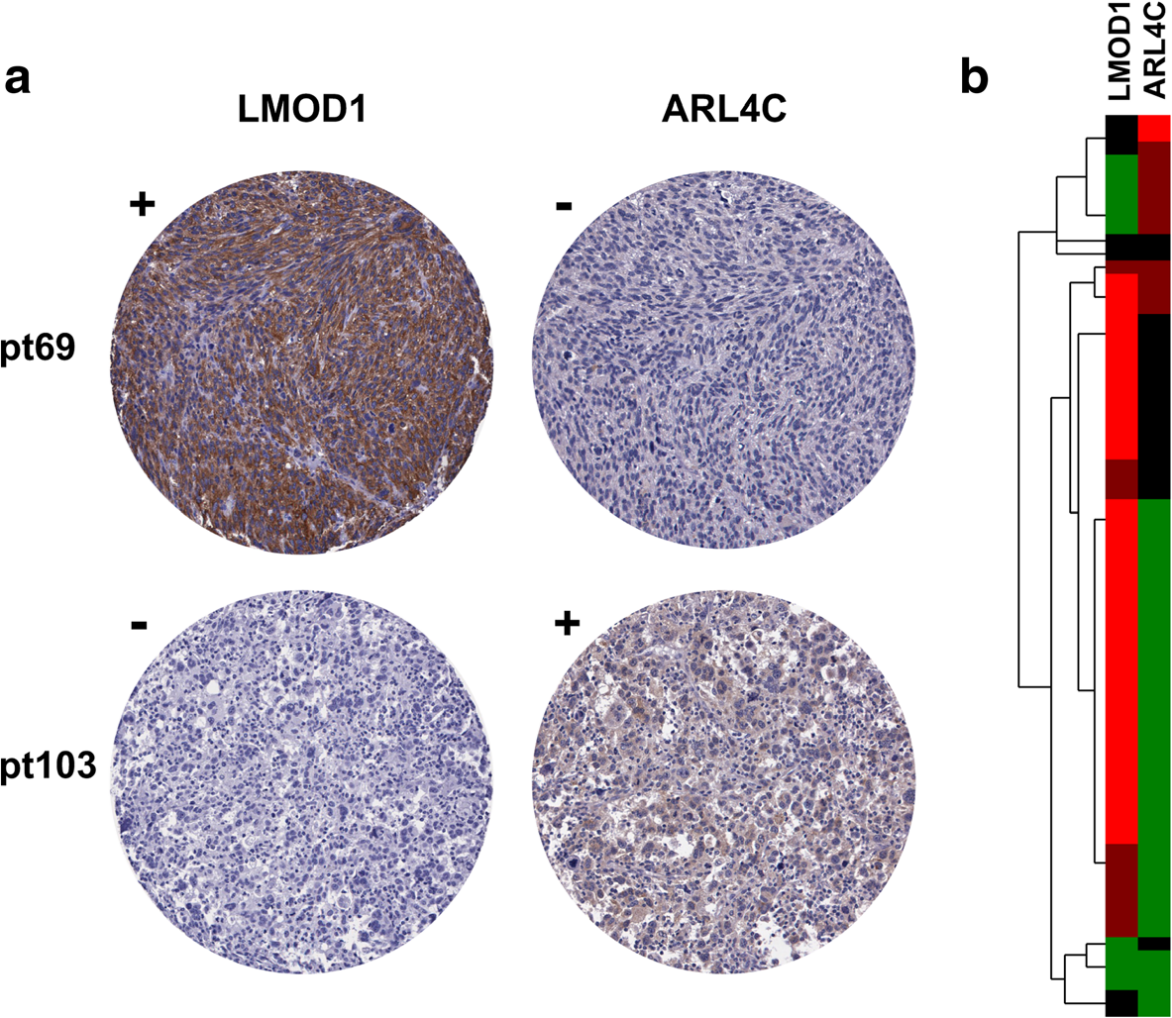
Immunohistochemistry staining of ULMS subtype specific biomarkers. **a** Representative staining of LMOD1 and ARL4C for a subtype I ULMS case (case # pt69) and a subtype II ULMS case (case # pt103). **b** Heatmap of LMOD1 and ARL4C IHC staining results on 68 ULMS cases. *Bright-red* and *dull-red* represent strong and weak staining, while *gree*n and *black* indicated negative and equivocal staining

To further validate ULMS subtypes and develop subtype specific diagnostic biomarkers, we selected ULMS subtype specific genes for IHC staining by SAM-seq result and availability of commercial antibodies. The IHC results showed that subtype I biomarker LMOD1 was positively stained in 51 of 68 ULMS cases (75%), while 13 subtype II ULMS cases were positive for ARL4C (13/68, 19%) (Fig. 4a), and the correlation coefficient of staining results of these two genes across all the ULMS cases is −0.43 (Fig. 4b).

## Discussion

Uterine sarcomas are composed of leiomyosarcoma, endometrial stromal sarcoma and carcinosarcoma. Among these, leiomyosarcoma is the most common subclass, mainly found in postmenopausal women [1, 23]. Although early diagnosis could improve the survival rate of ULMS patients, there are still challenges for treating late stage ULMS patients due to its high invasiveness and relatively high resistance to radiotherapy and chemotherapy [24]. Molecular subtyping of tumors based on their gene expression profiling have guided subtype-specific diagnosis, prognosis, and aided to develop subtype targeted therapies [17]. In our study, we identified two molecular subtypes of ULMS and found that these two subtypes exhibited significantly different gene expression patterns and distinct sensitivities to chemotherapy treatment. Nevertheless, please kindly noted that the number of patients with clinical treatment response information in this study is low, and additional large scale validation of treatment responses on distinct molecular subtypes will be needed.

Among the genes and pathways enriched, subtype I ULMS showed overexpression of smooth muscle-specific markers, including *LMOD1*, *SLMAP*, *MYLK*, *MYH11*. LMOD1, also known as Leiomodin-1, could be activated by serum response factor (SRF) or myocardin (MYOCD) and functions in smooth muscle cell differentiation [20]. SLMAP, or sarcolemmal membrane-associated protein, is involved in microtubule organization [25], excitation-contraction coupling [26] and myoblast fusion [27]. Myosin light chain kinase and Myosin-11 protein, encoded by *MYLK* and *MYH11*, respectively, are components of smooth muscle cells SMC contractile apparatus [28]. MYLK is a Ca^2+^/CaM-dependent kinase and involved in smooth muscle contraction by promoting the interaction between myosin and actin filaments [29]. Myosin-11 belongs to the myosin heavy chain family, and has contractile activity by hydrolyzing ATP [30].

Subtype II ULMS showed overexpression of genes enriched in pathways including cancer, TGF-β and Hedgehog signaling. Particularly, over-expression was found in the following genes, including *CDK6, MAPK13* and *HOXA1*. *CDK6* (Cyclin-dependent kinase 6) is a cell cycle regulator and forms a complex with cyclin D to initiate G1 to S phase transition by phosphorylating and inactivating Rb [31–33]. *MAPK13* (Mitogen-activated protein kinase 13) belongs to the MAP kinase family and functions in cell proliferation, differentiation and development. *MAPK13* is also involved in cell motility and invasion, serving as a diagnostic marker for cholangiocarcinoma [34]. *MAPK13* is highly expressed in uterine and ovary tumor tissues, especially in gynecological cancer stem cells, and has tumor-initiating activity that was involved in tumorigenesis [35]. Highly expressed in many types of tumor cells, *HOXA1* (Homeobox A1) is a DNA-binding protein and involved in facilitating cell proliferation, invasion, metastasis, and tumor progression [36, 37]. These proto-oncogenes were all over-expressed in subtype II, suggesting that the extent of malignancy of subtype II ULMS may be higher than that of subtype I, and subtype II may represent high-grade ULMS.

## Conclusions

In conclusion, we characterized distinct intrinsic molecular subtypes of ULMS with different gene signatures. Our findings provide new insights into the understanding of the pathogenesis of ULMS, facilitate the development of subtype-specific diagnostic biomarkers and targeted treatment for ULMS. Furthermore, our finding may provide valuable information to develop individualized medicine for ULMS patients.

## Additional files

Additional file 1: Figure S1. Delineation of two distinct molecular subtypes of ULMS. (A) Empirical cumulative distribution plots. (B) The increased area under the CDF curve along with increased number of molecular subtypes. (TIFF 215 kb)

Additional file 2: Figure S2. Heatmap of the top 500 genes over-expressed in distinct molecular subtypes. Based on SAM-seq result, the top 500 genes over-expressed in each subtype were selected to form the TOP500 genes. Hierarchical clustering of the TOP500 genes was performed by Cluster 3.0 using centroid linkage method. Each row denotes a gene and each column corresponds to a case of ULMS. Red, over-expressed genes; Green, down-expressed genes. (TIFF 2173 kb)

## Abbreviations

CDK6: Cyclin-dependent kinase 6
EMT: Epithelial to mesenchymal transition
GSEA: Gene Set Enrichment Analysis
HOXA1: Homeobox A1
IHC: Immunohistochemistry
LMOD1: Leiomodin-1
LMS: Leiomyosarcoma
MAP K13: Mitogen-activated protein kinase 13
MYH11: Myosin-11
MYLK: Myosin light chain kinase
MYOCD: Myocardin
SAM: Significance Analysis of Microarrays
SLMAP: Sarcolemmal membrane-associated protein
SRF: Serum response factor
TCGA: The Cancer Genome Atlas
ULMS: Uterine leiomyosarcoma

## References

[1] Miyata T, Sonoda K, Tomikawa J, Tayama C, Okamura K, Maehara K (2015). Genomic, Epigenomic, and Transcriptomic profiling towards identifying Omics features and specific biomarkers that distinguish uterine Leiomyosarcoma and Leiomyoma at molecular levels. Sarcoma.

[2] Gauthe M, Testart Dardel N, Nascimento C, Trassard M, Banal A, Alberini JL (2017). Uterine leiomyosarcoma metastatic to thyroid shown by 18F-FDG PET/CT imaging. Revista espanola de medicina nuclear e imagen molecular.

[3] Dandapani M, Seagle BL, Abdullah A, Hatfield B, Samuelson R, Shahabi S (2015). Metastatic uterine Leiomyosarcoma involving bilateral ovarian Stroma without capsular involvement implies a local route of Hematogenous dissemination. Case Rep Obstet Gynecol.

[4] Artioli G, Borgato L, Calamelli S, Azzarello G. Unusual cardiac metastasis of uterine leiomyosarcoma: case report and literature review. Tumori. 2016;102(Suppl. 2):90–2. 10.5301/tj.5000498 27079906

[5] Bogani G, Fuca G, Maltese G, Ditto A, Martinelli F, Signorelli M (2016). Efficacy of adjuvant chemotherapy in early stage uterine leiomyosarcoma: a systematic review and meta-analysis. Gynecol Oncol.

[6] Ducie JA, Leitao MM (2016). The role of adjuvant therapy in uterine leiomyosarcoma. Expert Rev Anticancer Ther.

[7] Monti STP, Mesirov J, Golub T (2003). Consensus clustering: a Resampling-based method for class discovery and visualization of gene expression microarray data. Mach Learn.

[8] Zivanovic O, Jacks LM, Iasonos A, Leitao MM, Soslow RA, Veras E (2012). A nomogram to predict postresection 5-year overall survival for patients with uterine leiomyosarcoma. Cancer.

[9] Bertucci F, Finetti P, Rougemont J, Charafe-Jauffret E, Cervera N, Tarpin C (2005). Gene expression profiling identifies molecular subtypes of inflammatory breast cancer. Cancer Res.

[10] Sorlie T, Perou CM, Tibshirani R, Aas T, Geisler S, Johnsen H (2001). Gene expression patterns of breast carcinomas distinguish tumor subclasses with clinical implications. Proc Natl Acad Sci U S A.

[11] Lei Z, Tan IB, Das K, Deng N, Zouridis H, Pattison S (2013). Identification of molecular subtypes of gastric cancer with different responses to PI3-kinase inhibitors and 5-fluorouracil. Gastroenterology.

[12] Cristescu R, Lee J, Nebozhyn M, Kim KM, Ting JC, Wong SS (2015). Molecular analysis of gastric cancer identifies subtypes associated with distinct clinical outcomes. Nat Med.

[13] An Y, Wang H, Jie J, Tang Y, Zhang W, Ji S (2017). Identification of distinct molecular subtypes of uterine carcinosarcoma. Oncotarget.

[14] Goldhirsch A, Wood WC, Coates AS, Gelber RD, Thurlimann B, Senn HJ (2011). Strategies for subtypes--dealing with the diversity of breast cancer: highlights of the St. Gallen international expert consensus on the primary therapy of early breast cancer 2011. Ann Oncol.

[15] Guo X, Jo VY, Mills AM, Zhu SX, Lee CH, Espinosa I (2015). Clinically relevant molecular subtypes in Leiomyosarcoma. Clin Cancer Res.

[16] Italiano A, Lagarde P, Brulard C, Terrier P, Lae M, Marques B (2013). Genetic profiling identifies two classes of soft-tissue leiomyosarcomas with distinct clinical characteristics. Clin Cancer Res.

[17] Wilkerson MD, Hayes DN (2010). ConsensusClusterPlus: a class discovery tool with confidence assessments and item tracking. Bioinformatics.

[18] Peter RJ (1987). Silhouettes: a graphical aid to the interpretation and validation of cluster analysis. J Comput Appl Math.

[19] Hung JH, Yang TH, Hu Z, Weng Z, DeLisi C (2012). Gene set enrichment analysis: performance evaluation and usage guidelines. Brief Bioinform.

[20] Nanda V, Miano JM (2012). Leiomodin 1, a new serum response factor-dependent target gene expressed preferentially in differentiated smooth muscle cells. J Biol Chem.

[21] Yeung KK, Bogunovic N, Keekstra N, Beunders AA, Pals J, van der Kuij K (2017). Transdifferentiation of human dermal fibroblasts to smooth muscle-like cells to study the effect of MYH11 and ACTA2 mutations in aortic aneurysms. Hum Mutat.

[22] Okumu LA, Bruinton S, Braden TD, Simon L, Goyal HO (2012). Estrogen-induced maldevelopment of the penis involves down-regulation of myosin heavy chain 11 (MYH11) expression, a biomarker for smooth muscle cell differentiation. Biol Reprod.

[23] Skorstad M, Kent A, Lieng M (2016). Uterine leiomyosarcoma - incidence, treatment, and the impact of morcellation. A nationwide cohort study. Acta Obstet Gynecol Scand.

[24] Samarnthai N, Hall K, Yeh IT (2010). Molecular profiling of endometrial malignancies. Obstet Gynecol Int.

[25] Guzzo RM, Sevinc S, Salih M, Tuana BS (2004). A novel isoform of sarcolemmal membrane-associated protein (SLMAP) is a component of the microtubule organizing centre. J Cell Sci.

[26] Nader M, Westendorp B, Hawari O, Salih M, Stewart AF, Leenen FH (2012). Tail-anchored membrane protein SLMAP is a novel regulator of cardiac function at the sarcoplasmic reticulum. Am J Physiol Heart Circ Physiol.

[27] Guzzo RM, Wigle J, Salih M, Moore ED, Tuana BS (2004). Regulated expression and temporal induction of the tail-anchored sarcolemmal-membrane-associated protein is critical for myoblast fusion. Biochem J.

[28] Renard M, Callewaert B, Baetens M, Campens L, MacDermot K, Fryns JP (2013). Novel MYH11 and ACTA2 mutations reveal a role for enhanced TGFbeta signaling in FTAAD. Int J Cardiol.

[29] Yin F, Hoggatt AM, Zhou J, Herring BP (2006). 130-kDa smooth muscle myosin light chain kinase is transcribed from a CArG-dependent, internal promoter within the mouse mylk gene. Am J Physiol Cell Physiol.

[30] Kuang SQ, Kwartler CS, Byanova KL, Pham J, Gong L, Prakash SK (2012). Rare, nonsynonymous variant in the smooth muscle-specific isoform of myosin heavy chain, MYH11, R247C, alters force generation in the aorta and phenotype of smooth muscle cells. Circ Res.

[31] Meyerson M, Harlow E (1994). Identification of G1 kinase activity for cdk6, a novel cyclin D partner. Mol Cell Biol.

[32] Bertoli C, Skotheim JM, de Bruin RA (2013). Control of cell cycle transcription during G1 and S phases. Nat Rev Mol Cell Biol.

[33] Ezhevsky SA, Ho A, Becker-Hapak M, Davis PK, Dowdy SF (2001). Differential regulation of retinoblastoma tumor suppressor protein by G(1) cyclin-dependent kinase complexes in vivo. Mol Cell Biol.

[34] Tan FL, Ooi A, Huang D, Wong JC, Qian CN, Chao C (2010). p38delta/MAPK13 as a diagnostic marker for cholangiocarcinoma and its involvement in cell motility and invasion. Int J Cancer.

[35] Yasuda K, Hirohashi Y, Kuroda T, Takaya A, Kubo T, Kanaseki T (2016). MAPK13 is preferentially expressed in gynecological cancer stem cells and has a role in the tumor-initiation. Biochem Biophys Res Commun.

[36] Yuan C, Zhu X, Han Y, Song C, Liu C, Lu S (2016). Elevated HOXA1 expression correlates with accelerated tumor cell proliferation and poor prognosis in gastric cancer partly via cyclin D1. J Exp Clin Cancer Res.

[37] Wang H, Liu G, Shen D, Ye H, Huang J, Jiao L (2015). HOXA1 enhances the cell proliferation, invasion and metastasis of prostate cancer cells. Oncol Rep.

