# Supplementary figures and images for: Distinct molecular subtypes of uterine leiomyosarcoma respond differently to chemotherapy treatment
Source: BMC Cancer. 2017 Sep 11;17:639. doi: 10.1186/s12885-017-3568-y (PMC5594508; doi:10.1186/s12885-017-3568-y)

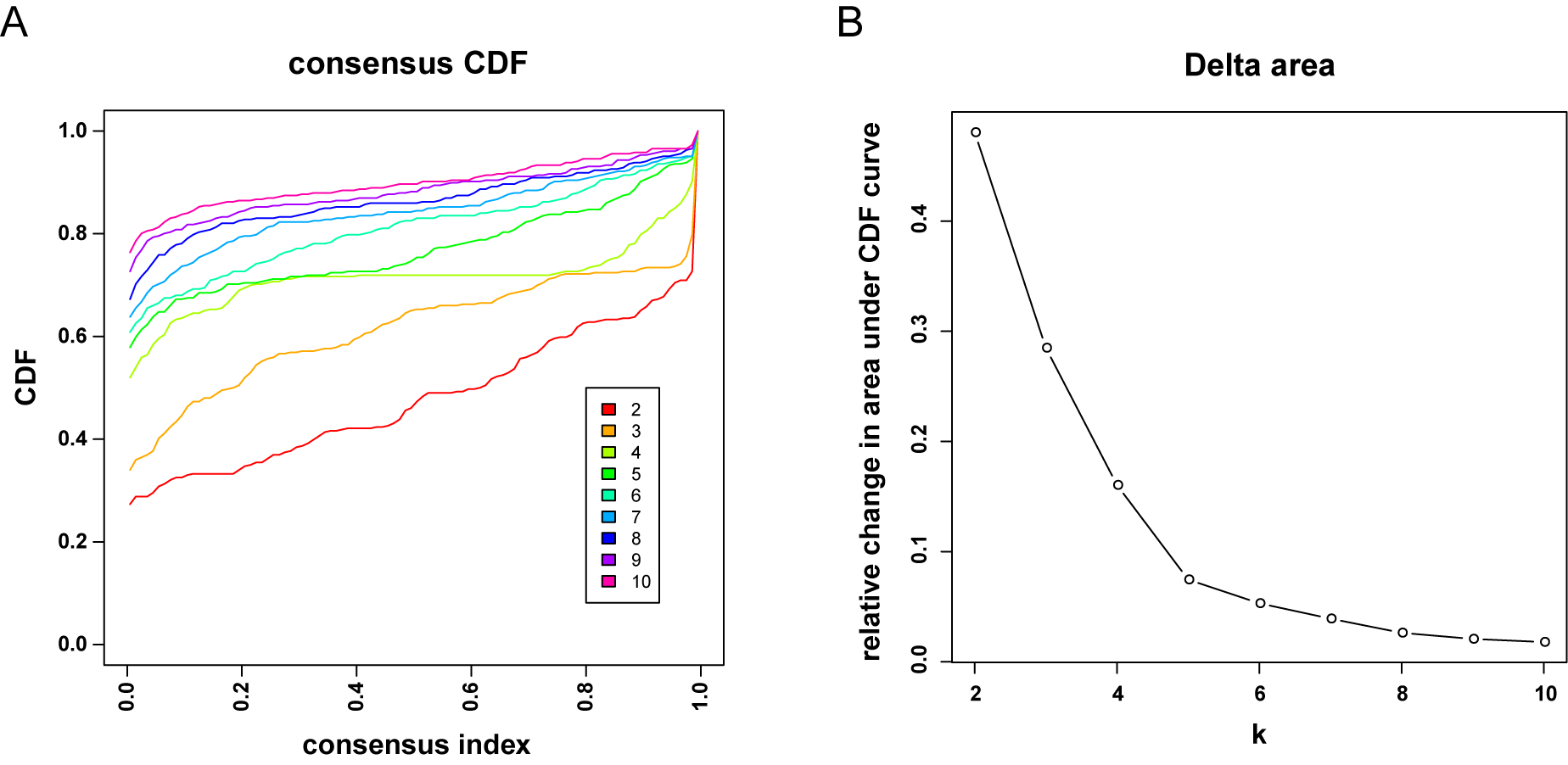

Supplement: Supplementary file 1 — Delineation of two distinct molecular subtypes of ULMS. (A) Empirical cumulative distribution plots. (B) The increased area under the CDF curve along with increased number of molecular subtypes. (TIFF 215 kb) [file 12885_2017_3568_MOESM1_ESM.tif]

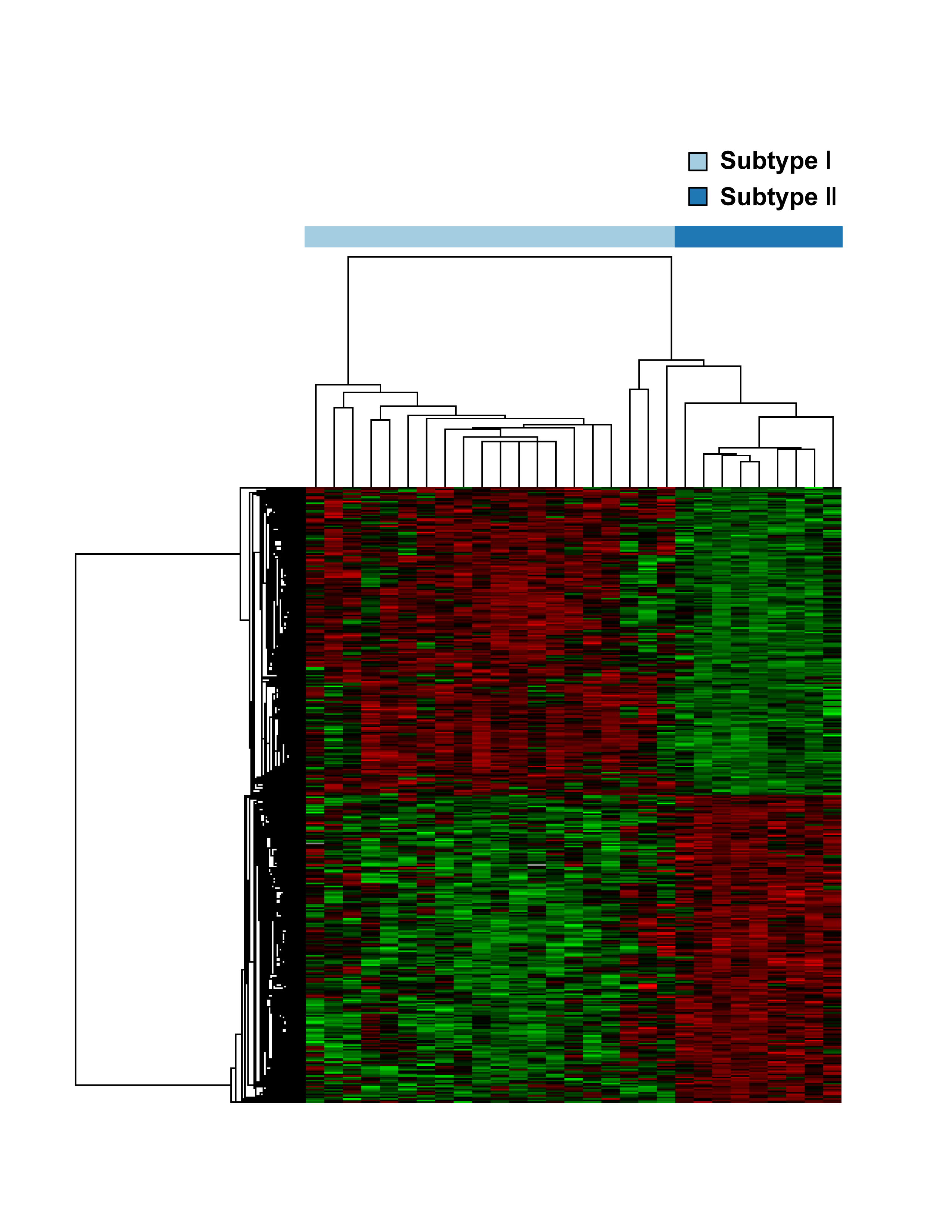

Supplement: Supplementary file 2 — Heatmap of the top 500 genes over-expressed in distinct molecular subtypes. Based on SAM-seq result, the top 500 genes over-expressed in each subtype were selected to form the TOP500 genes. Hierarchical clustering of the TOP500 genes was performed by Cluster 3.0 using centroid linkage method. Each row denotes a gene and each column corresponds to a case of ULMS. Red, over-expressed genes; Green, down-expressed genes. (TIFF 2173 kb) [file 12885_2017_3568_MOESM2_ESM.tif]
